# 2024: anticipating record-breaking performances in front crawl swimming through mathematical analysis

**DOI:** 10.3389/fphys.2024.1397946

**Published:** 2024-06-05

**Authors:** Aurélien Patoz, Louis Finiel, Fabio Borrani

**Affiliations:** ^1^ Institute of Sport Sciences, University of Lausanne, Lausanne, Switzerland; ^2^ Volodalen Swiss SportLab, Research and Development Department, Aigle, Switzerland

**Keywords:** swimming performance, record prediction, 2024 olympic games, performance modelling, front crawl swimming

## Abstract

**Introduction:** This study presented a novel approach to predict future front crawl swimming world records (WRs) by employing a methodology that integrated performance data from both running and front crawl swimming.

**Methods:** By extracting the top one running and swimming performances from 1995 to 2023 and applying a model that correlates physiological characteristics such as maximum aerobic power, anaerobic capacity, the decrement in maximum power with prolonged effort, and performance speed and duration, it was possible to project the potential record-breaking performances in 2024 across various swimming distances for both male and female athletes. Furthermore, this approach was expected to be less susceptible to the influence of the full-body swimsuit era, which may have disrupted the typical trajectory of swimming performance progression.

**Results:** The average relative error between the top one and estimated speeds in front crawl swimming (50–1,500 m, from 1995 to 2023, and for male and female) was 0.56% ± 0.17%. For male, WR in longer distances have been predicted with new WR in the 400 and 800 m. A more ambitious prediction was noted among female, with twice as many WR as among male illustrated by new WR in the 50, 200, 400 and 800 m.

**Discussion:** This study illustrated that the utilization of a prediction model based on physiological parameters yielded plausible time estimates. Additionally, the research accentuated the ongoing trajectory of surpassing existing WR into 2024, illustrating the competitive zeal fueled by an emerging framework of exceptional swimmers.

## Introduction

Among sports, swimming has demonstrated a particularly rapid evolution of world records (WRs) in recent years (Scheerder et al., 2011). Notably, 40% of male’s WR and 55% of female’s WR have been broken since 2020 (World aquatics, 2022). However, the scientific literature has predominantly focused on disciplines like running, with publications often outnumbering those on swimming by a ratio of up to 4:1 (Ben-Zaken et al., 2022). Sport predictions are useful for optimizing training and strategy planning, thereby aiding athletes and coaches in achieving their goals. Additionally, they offer valuable insights into performance trends and dynamics in sports (Mujika et al., 2023).

When it comes to performance prediction, studies by authors like Hopkins (2000) suggested a decade increase of 1%–1.5% in running performance, implying limitless improvement potential, whereas others such as Peronnet and Thibault (1989) proposed inherent performance limits. In swimming, multiples models have been developed (Mujika et al., 2023) and various statistical models, including extreme value theory (Spearing et al., 2021), neural network models (Maszczyk et al., 2012), and nonlinear regression models (Heazlewood and Walsh, 2011), have been employed to predict athletic performance. Recently, Wu et al. (2022) extended a nonlinear regression model to a Bayesian framework for predicting winning times in individual swimming events at the 2021 and 2024 Olympic Games. Most of the models in swimming performance prediction have traditionally relied on extrapolating past performance times.

Recently, Wu et al. (2022) extended a nonlinear regression model to a Bayesian framework for predicting winning times in individual swimming events at the 2021 and 2024 Olympic Games. Most of the models in swimming performance prediction have traditionally relied on extrapolating past performance times.

Swimming could present unique challenges for predictive modelling due to frequent rule changes, notably the well-known regulation of full-body swimsuits, which arrived on the market in the late 1990s and led to a surge in WR broken between 2008 and 2009. O'Connor L and Vozenilek (2011) concluded that full body swimsuits were at the origin of the urge number of broken records in 2009 by notably comparing this evolution with running. Unlike in other sports, this regulatory shift might have disrupted the typical progression of performance and could make predictions based solely on time performance more limited (Spearing et al., 2021).

Numerous mechanical and physiological models have been formulated to describe the processes that underline human performance. Moreover, swimming prediction models considering the underlying physiological attributes of athletes is not well referenced in the literature in contrast to running, where there exists a well-established relationship between metabolic power and sustained speed (di Prampero, 1986). Nonetheless, fundamental principles of human energetics have been elegantly captured in mathematical formulations (Peronnet and Thibault, 1989), delineating the energy expenditure rate as a function of key physiological characteristics such as maximal aerobic power (MAP), anaerobic capacity (*A*), and the decrement in maximum power with prolonged effort (*E*).

Yet swimming and running share many physiological characteristics. During submaximal exercise, variables including cardiac output, stroke volume, heart rate and arteriovenous oxygen difference exhibited comparable values between swimming and running modalities (Holmer, 1972; Holmer et al., 1974). Elite swimmers typically exhibit maximal oxygen consumption ( $\dot{V}$ O2max) ranging from 66 to 80 mL/kg/min (Maglischo, 2003), comparable to elite runners. At peak levels of activity, stroke volume during swimming was found to be equivalent to that during running and measurements of blood lactate and oxygen uptake from blood circulating in the exercising leg showed no difference between swimming and running at their maximal intensities (Holmér et al., 1974). Swimming events ranging from 50 to 1500 m align with running distances of 100–5,000 m in terms of exertion duration. Despite distinctive techniques, elite swimmers and middle-distance runners (200–800 m) exhibit comparable levels of endurance and power during exertions of equivalent duration. This equivalence can be found in their utilization of similar percentages of $\dot{V}$ O2max and maximal voluntary muscle contraction during performance (Al-Khelaifi et al., 2018). In addition, a study by O'Connor L and Vozenilek (2011) found similarities in sports performance (i.e., metabolic power developed for the same competition time), reinforcing the hypothesis that training methods are similar. Swimmers and runners may share equivalent physiological characteristics, underscoring a foundational similarity in their athletic profiles. However, the distinction must be made in the application of these shared physiological traits in the context of performance between the two sports. The discipline of swimming, characterized by movement in a horizontal plane through a more resistant medium, presents unique challenges that are absent in athletics. This distinct environment elevates the importance of optimizing propulsion efficiency and minimizing resistance to enhance performance. In contrast, running involves a different set of performance dynamics, where the engagement with these physiological traits does not encounter the same level of resistance-driven complexity.

In swimming, the assessment of energy expenditure entails consideration of multiple factors including speed, drag force, propelling efficiency, and gross efficiency (Toussaint and Truijens, 2005). Assuming equivalence in physiological characteristics between elite swimmers and runners for a given duration of effort, it becomes feasible to estimate these parameters or at least an encompassing value thereof, enabling the prediction of swimming race times. Therefore, the objective of this study was to compute the physiological attributes of athletes (MAP, *A* and *E*) for each year since 1995 and utilize them to determine swimming performance factors for predicting race times in the 2024 Olympic year.

## Materials and methods

### Data

To cover the range of swimming distances from 50 m to 1,500 m, running distances from 200 m to 5,000 m have been taken into account. The top one running performance of 200, 400, 800, 1,500, and 5,000 m and the top one front crawl swimming performance of 50, 100, 200, 400, 800, 1,500 m for male and female were extracted separately for all years and Olympic years between 1995 and 2023 using the following websites https://worldathletics.org/records/all-time-toplists and https://www.worldaquatics.com/swimming/rankings for running and swimming, respectively. This corresponds to five running performances and six swimming performances for male and female each year. Hence, this corresponds to 145 running performances for all years and 35 running performances for Olympic years for male and female. As for swimming, this corresponds to 174 and 42 performances for all years and Olympic years, respectively, for male and female. The top one performance, i.e., a single value for each combination of distance, year, and gender, were extracted to make sure that each predicting race time represented the best possible result.

### Experimental design

For each year, the model physiological characteristics (MAP, *A* and *E*) of a prototypical athlete capable of achieving the top one running race times over distances ranging from 200 to 1500 m were calculated. These characteristics were used to calculate the metabolic power developed for a given effort duration over different distances in front crawl swimming (50, 100, 200, 400, 800, and 1,500 m). From these powers, parameters encompassing the multiple factors of swimming performance, namely speed, drag force, and propulsive and gross efficiencies (Toussaint et al., 2000) were calculated (*K* and *n* in what follows). The physiological characteristics and swimming performance parameters (MAP, *A*, *E*, *K*, and *n*) were linearly evaluated over the years to obtain their prediction values for 2024. This finally allowed to predict the 2024 best time performances for front crawl swimming for 50, 100, 200, 400, 800, 1,500 m for male and female.

### Theoretical considerations

#### Running

The average metabolic power output (*P*) required to run at a given speed (*v*) can be computed following the equation developed by di Prampero (1984):

$$P\left(v\right)=\text{BMR}+3.86v+\frac{0.4\;\text{BSA}}{m}v^{3}+\frac{2}{d}v^{3},$$ (1)

where BSA and BMR stand for body surface area and basal metabolic rate, respectively, *m* is the body mass, and *d* the running distance. BMR is set to 1.2 W/kg (Peronnet and Thibault, 1989) and BSA and *m* are set to 1.8 m^2^ and 70 kg for male, and to 1.6 m^2^ and 50 kg for female (Peronnet and Thibault, 1989).

Another model, developed by Peronnet and Thibault (1989), is based on the postulate that *P* is the sum of aerobic and anaerobic powers and is determined from physiological characteristics. This model of running performance gives *P* as function of the running duration (*t*) and is described by the following equation:

$$P\left(t\right)=\text{BMR}+B\left[1+\frac{k_{1}}{t}\left(e^{-\frac{t}{k_{1}}}-1\right)\right]+\frac{S}{t}\left(1-e^{-\frac{t}{k_{2}}}\right).$$ (2)

Briefly, $k_{1}$ and $k_{2}$ are time constants for the kinetics of aerobic and anaerobic metabolism at the beginning of exercise and are equal to 30 s (Linnarsson, 1974; Hagberg et al., 1978; Fox et al., 1980) and 20 s (Peronnet and Thibault, 1989), respectively. *B* is the relative maximal aerobic power [difference between MAP and BMR] and *S* is the energy from anaerobic metabolism available to a runner over time. For *B* and *S*, athletes are only able to maintain their maximum intensity for a given duration ( $t_{MAP}$ ) of ∼420 s (Costill and Fox, 1969; Londeree, 1986). After this period, relative maximal aerobic power, and the amount of energy available through anaerobic metabolism progressively decrease according to ln⁡t as race duration increases.

Hence, for $t\leq t_{MAP}$ (Costill and Fox, 1969; Gollnick and Hermansen, 1973; Londeree, 1986):

B=MAP−BMR

S=A

and, for $t>t_{MAP}$ (Costill and Fox, 1969; Gollnick and Hermansen, 1973; Londeree, 1986):

$$B=\text{MAP}-\text{BMR}+E\;\ln\left(\frac{t}{t_{MAP}}\right)$$

$$S=A\left[1+f\;\ln\left(\frac{t}{t_{MAP}}\right)\right]$$

*f* represents the rate of decline of *S* with running duration when running duration is larger than $t_{MAP}$ and is equal to −0.233 (Gollnick and Hermansen, 1973).

#### Front crawl swimming

When the swimmer is moving at a constant speed, according to Newton’s second law, the swimmer’s propulsive force (*F*_*p*_) is equal to the active drag force (*F*_*d*_). The relationship between this same active drag force and speed (*v*) has been defined by a power function (Eq. 3) (di Prampero, 1986; Toussaint et al., 2004):

$$F_{d}\left(v\right)=av^{n},$$ (3)

where *a* and *n* are the parameters of the power function. The total useful mechanical power output (*P*_*u*_) is equal to the product of *F*_*p*_ and *v*:

$$P_{u}\left(v\right)=F_{p}\left(v\right)v=F_{d}\left(v\right)v=av^{n+1}.$$ (4)

It is important to acknowledge that the mechanical power (P_m_) is not entirely converted into useful propulsive power (P_u_). This conversion efficiency is quantified by the propelling efficiency (ε_p_), which is defined as the ratio of these two powers:

$$\varepsilon_{p}=\frac{P_{u}\left(v\right)}{P_{m}\left(v\right)}$$

leading to:

$$P_{m}\left(v\right)=\frac{P_{u}\left(v\right)}{\varepsilon_{p}},$$ (5)

It should also be considered that only a fraction of the metabolic power (*P*) is transformed into *P*_*m*_. The rest is converted into heat and to support other bodily functions. Power metabolic alteration is described by gross efficiency ( $\varepsilon_{g}$ ):

$$\varepsilon_{g}=\frac{P_{m}\left(v\right)}{P\left(v\right)}$$

leading to:

$$P\left(v\right)=\frac{P_{m}\left(v\right)}{\varepsilon_{g}}.$$ (6)

By substituting Eqs 4, 5 into Eq. 6, one could obtain:

$$P\left(v\right)=\frac{a}{\varepsilon_{g}\varepsilon_{p}}v^{n+1}.$$ (7)

Since the parameters *a*, $\varepsilon_{g}$ , and $\varepsilon_{p}$ are constants, they can be replaced by a parameter encompassing them (*K*)*,* leading to:

$$P\left(v\right)=Kv^{n+1}.$$ (8)

This equation Eq. 8) contains two parameters, a coefficient *K* and an exponent *n*.

### Data analysis

Using v=d/t and equating Eqs 1, 2, the optimal set of *A*, MAP, and *E* was obtained for each year by minimizing the relative running error ( $e_{r}$ ) between the durations of the top one running performances ( $t_{r}$ ) obtained from the world athletics website and the estimated durations of these performances ( $t_{e}$ ) according to:

$$e_{r}=\frac{1}{N}\sum_{i=1}^{N}\frac{\left|t_{e,i}-t_{r,i}\right|}{t_{r,i}},$$ (9)

where *N* is the number of top one running performance. At this step, a prototypical athlete with standardized BMR of 1.2 W/kg, weight of 70 kg for male and 60 kg for female, and body surface area of 1.80 m^2^ for male and 1.60 m^2^ for female was created for each year.

As front crawl swimmers were assumed to develop similar metabolic power than runners for a given effort duration, Eq. 2 with the optimal set of *A*, MAP, and *E* could be used to describe the average metabolic power of front crawl swimmers using the corresponding effort durations (for the same prototypical athlete) for each year.

At this point, using t=d/v and equating Eqs 2, 8, the optimal set of *K* and *n* was obtained for each year by minimizing the relative swimming error ( $e_{s}$ ) between the speeds of the top one front crawl swimming performances ( $v_{s}$ ) obtained from the world aquatics website and the estimated speeds of these performances ( $v_{e}$ ) according to

$$e_{s}=\frac{1}{N}\sum_{i=1}^{N}\frac{\left|v_{e,i}-v_{s,i}\right|}{v_{s,i}},$$ (10)

where *N* is the number of top one swimming performance.

The variation of *A*, MAP, and *E* as well as the variation of *K* and *n* were linearly evaluated over the years to obtain prediction values for 2024 for male and female considering all years and the Olympic years separately. Each 2024 prediction was given by the extrapolated value of the linear relation between the given variable and years if this relationship was significant, and by the average value of this variable over the years if this relationship was not significant.

Finally, this allowed predicting the 2024 best performances for each front crawl swimming distance (50, 100, 200, 400, 800, and 1500 m). These 2024 predictions were obtained by: 1) equating Eqs 2, 8 using the 2024 predictions of *A*, MAP, *E*, *K*, and *n* and 2) solving for *t* using v=d/t . Data analysis was performed using Python (v3.8.16, retrieved from http://www.python.org).

### Statistical analysis

Descriptive statistics are presented using mean ± standard deviation. To be able to perform a linear regression between years and any of the optimal variables among *A*, MAP, *E*, *K*, and *n,* several conditions must be fulfilled. Hence, the independence of the residuals, normality of the residuals, and homogeneity of the variance of the residuals were tested using Durbin-Watson, Shapiro-Wilk and Breusch-Pagan tests, respectively. As a rule of thumb, a value between 1 and 3 of the Durbin-Watson statistics is considered as relatively normal, meaning that the residuals are independent. Then, the linearity between years and any of *A*, MAP, *E*, *K*, and *n* was evaluated using Pearson’s correlation coefficient (*r*) together with its corresponding *p*-value. Statistical analysis was performed using Python with a level of significance set at *p* ≤ 0.05.

## Results

The relative running errors reported to obtain the optimal set of MAP, *A*, and *E* (Eq. (9)) were 0.45% ± 0.30% for male and 0.45% ± 0.23% for female for all years and 0.46% ± 0.26% for male and 0.48% ± 0.34% for female for Olympic years.

As for running data for male and female and considering all years and Olympic years, the residuals were independent, i.e., the Durbin-Watson statistics ranged between 1.2 and 3.0, and normality and homogeneity of residuals were satisfied (0.07 ≤ *p* ≤ 0.99). Hence, these results allowed us to perform the linear regression.

**FIGURE 1:**
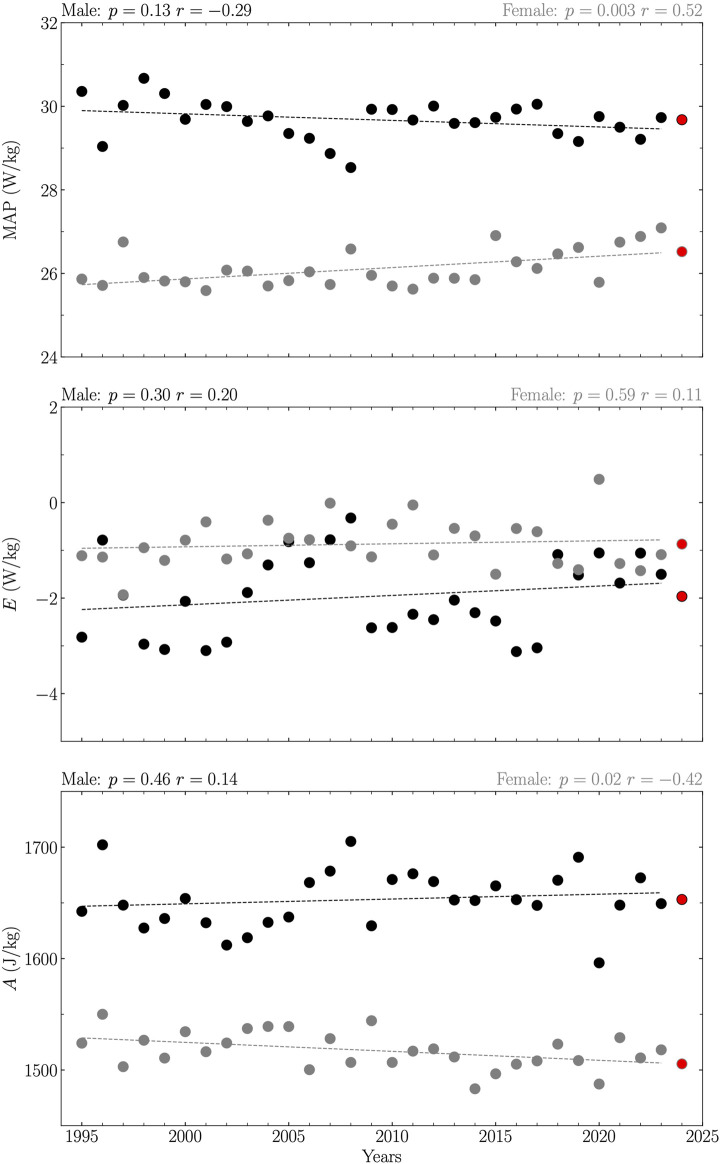
Maximal Aerobic Power (MAP), reduction in maximum power that occurs with increasing running duration (*E*), and capacity of anaerobic metabolism (*A*) obtained from the top one running performances of 200, 400, 800, 1,500, and 5,000 m for all years between 1995 and 2023 together with their 2024 predictions. Data are reported separately for male (black circles and line) and female (gray circles and line). Each prediction (red circle) was given by the extrapolated value of the linear relation between the given variable and years if this relationship was significant, and by the average value of this variable over the years otherwise. The linearity was evaluated using Pearson’s correlation coefficient (*r*) together with its corresponding *p*-value and considered significant if *p* ≤ 0.05.

**FIGURE 2:**
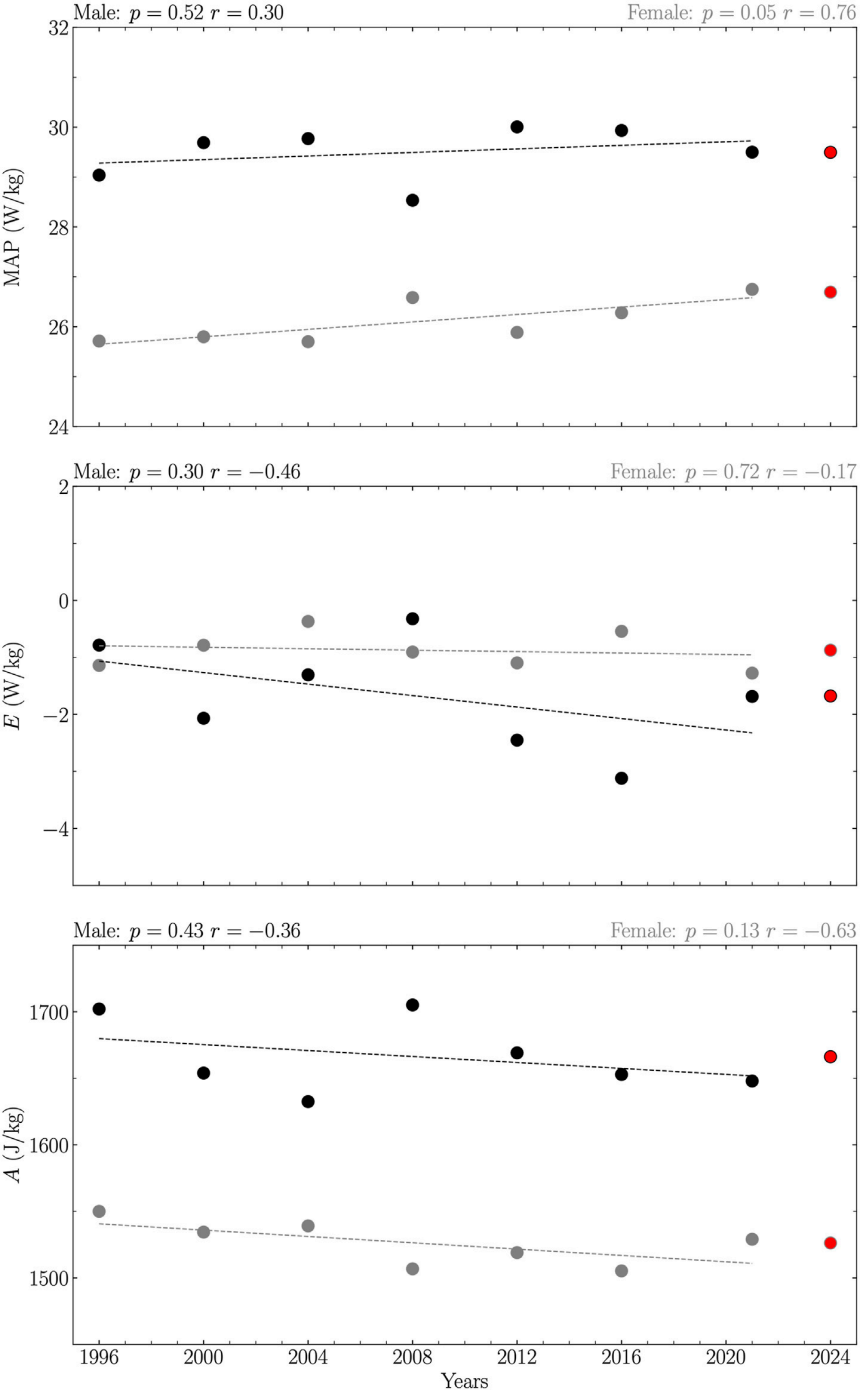
Maximal Aerobic Power (MAP), reduction in maximum power that occurs with increasing running duration (*E*), and capacity of anaerobic metabolism (*A*) obtained from the top one running performances of 200, 400, 800, 1,500, and 5,000 m for Olympic years between 1995 and 2023 together with their 2024 predictions. Data are reported separately for male (black circles and line) and female (gray circles and line). Each prediction (red circle) was given by the extrapolated value of the linear relation between the given variable and years if this relationship was significant, and by the average value of this variable over the years otherwise. The linearity was evaluated using Pearson’s correlation coefficient (*r*) together with its corresponding *p*-value and considered significant if *p* ≤ 0.05.

Analysis revealed a linear relationship between years and maximal aerobic power (MAP) and amplitude (*A*) across all years from 1995 to 2023 for females, indicating an increase in MAP over the years (MAP = 0.03 years – 28.59) and a decline in *A* (*A* = −0.81 years + 3133.99) with statistical significance (*p* ≤ 0.02; Figure 1). A similar linear increase in MAP with years (MAP = 0.04 years – 48.88) was observed for female athletes for the Olympic years within the same period, with statistical significance (*p* = 0.05; Figure 2). However, no significant correlations were found in other examined relationships (*p* ≥ 0.13; Figures 1, 2).

Predictions for MAP, *E* and *A* for the year 2024, considering both all years and Olympic years separately between 1995 and 2023 for male and female athletes, are summarized in Table 1.

**TABLE 1:** 2024 predictions of physiological characteristics.

Years	Sex	MAP (W/kg)	*E* (W/kg)	*A* (J/kg)
All	M	29.68	−1.96	1653.03
	F	**26.52**	−0.87	**1505.47**
Olympic	M	29.50	−1.68	1666.24
	F	**26.69**	−0.87	1526.20

Notes. Maximal Aerobic Power (MAP), reduction in maximum power that occurs with increasing running duration (*E*), and capacity of anaerobic metabolism (*A*) obtained from the top one running performances of 200, 400, 800, 1,500, and 5,000 m considering separately all years and Olympic years between 1995 and 2023 and male (M) and female (F). Each prediction was given by the extrapolated value of the linear relation between the given variable and years if this relationship was significant (*p* ≤ 0.05), and by the average value of this variable over the years otherwise. Predictions based on an extrapolated value are shown in bold.

The relative swimming errors reported to obtain the optimal set of *K*, and *n* (Eq. (10)) were 0.64% ± 0.20% for male and 0.35% ± 0.12% for female for all years and 0.82% ± 0.21% for male and 0.42% ± 0.15% for female for Olympic years.

As for swimming data for male and female and considering all years and Olympic years, the residuals were independent, i.e., the Durbin-Watson statistics ranged between 1.5 and 2.8, and normality and homogeneity of residuals were satisfied (0.11 ≤ *p* ≤ 0.97). Hence, these results allowed us to perform the linear regression.

**FIGURE 3:**
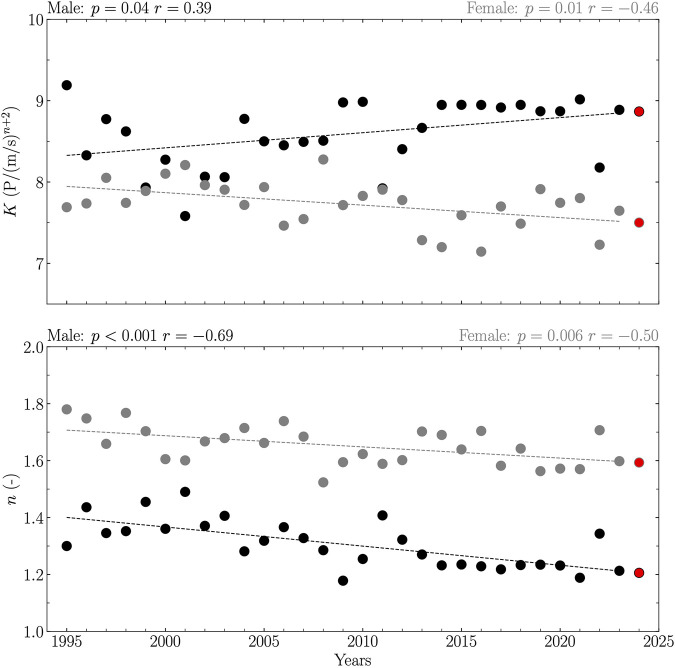
Coefficient (*K*) and exponent (*n*) obtained from the top one front crawl swimming performances of 50, 100, 200, 400, 800, and 1,500 m for all years between 1995 and 2023 together with their 2024 predictions. Data are reported separately for male (black circles and line) and female (gray circles and line). Each prediction (red circle) was given by the extrapolated value of the linear relation between the given variable and years if this relationship was significant, and by the average value of this variable over the years otherwise. The linearity was evaluated using Pearson’s correlation coefficient (*r*) together with its corresponding *p*-value and considered significant if *p* ≤ 0.05.

**FIGURE 4:**
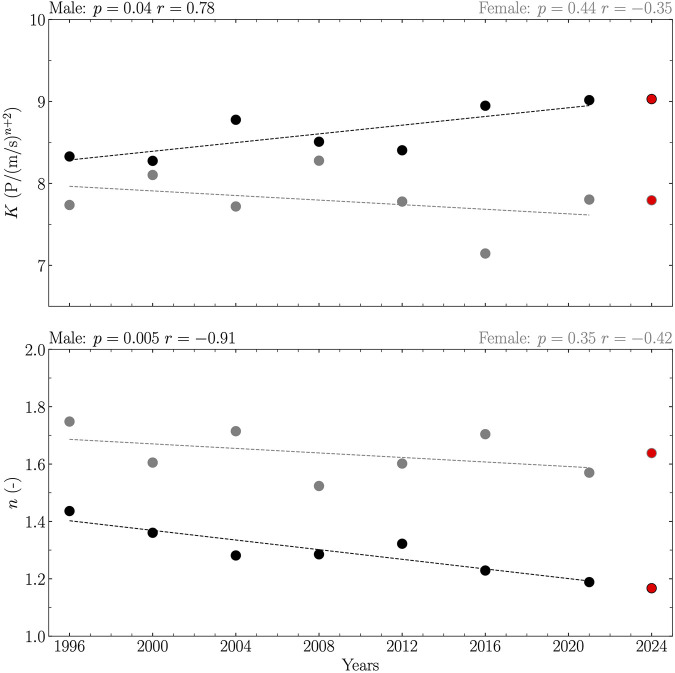
Coefficient (*K*) and exponent (*n*) obtained from the top one front crawl swimming performances of 50, 100, 200, 400, 800, and 1,500 m for Olympic years between 1995 and 2023 together with their 2024 predictions. Data are reported separately for male (black circles and line) and female (gray circles and line). Each prediction (red circle) was given by the extrapolated value of the linear relation between the given variable and years if this relationship was significant, and by the average value of this variable over the years otherwise. The linearity was evaluated using Pearson’s correlation coefficient (*r*) together with its corresponding *p*-value and considered significant if *p* ≤ 0.05.

When considering all years between 1995 and 2023, linearity was reported between years and both *K* and *n* for male and female (*p* ≤ 0.04; Figure 3), revealing an increase in *K* with years for male (*K* = 0.02 years – 29.80), a decrease in *K* with years for female (*K* = – 0.01 years + 38.56), and a decrease in *n* with years for male (*n* = – 0.01 years + 14.79) and female (*n* = – 0.00 years + 9.54). When considering Olympic years between 1995 and 2023, linearity was reported between years and *K* and *n* for male (*p* ≤ 0.04; Figure 4), revealing an increase in *K* with years (*K* = 0.03 years – 44.71) and a decrease in *n* with years (*n* = – 0.01 years + 18.17). The relations for female were not significant (*p* ≥ 0.35; Figure 4).

2024 predictions of *K* and *n* considering separately all years and Olympic years between 1995 and 2023 and male and female are summarized in Table 2.

**TABLE 2:** 2024 predictions of parameters encompassing the multiple factors of swimming performance.

Years	Sex	*K*	*n*
All	M	**8.87**	**1.21**
	F	**7.50**	**1.59**
Olympic	M	**9.03**	**1.17**
	F	7.79	1.64

Notes. Coefficient (*K*) and exponent (*n*) obtained from the top one front crawl swimming performances of 50, 100, 200, 400, 800, and 1,500 m considering separately all years and Olympic years between 1995 and 2023 and male (M) and female (F). Each prediction was given by the extrapolated value of the linear relation between the given variable and years if this relationship was significant (*p* ≤ 0.05), and by the average value of this variable over the years otherwise. Predictions based on an extrapolated value are shown in bold.

When considering all years, the 400 and 800 m front crawl swimming WRs are predicted to be broken for male (−0.26% and −0.04%, respectively; Table 3) as well as the 50, 200, 400, and 800 m for female (−0.91, −0.75, −0.37, and −0.04%, respectively; Table 3).

**TABLE 3:** 2024 best predicted time performances for front crawl swimming.

Sex	Performance	Duration (s)					
		50 m	100 m	200 m	400 m	800 m	1500 m
M	World record	20.91	46.86	102.00	220.07	452.12	871.02
	All years	20.95	46.88	103.98	**219.49**	**451.93**	878.42
	Olympic years	**20.72**	**46.51**	103.61	**219.26**	**451.93**	876.75
F	World record	23.61	51.71	112.85	235.38	484.79	920.48
	All years	**23.48**	51.77	**112.63**	**235.31**	**482.37**	923.79
	Olympic years	24.09	53.07	115.20	240.41	492.74	943.13

Notes. World records; world record at the end of 2023, all years; prediction with data from 1995 to 2023; Olympic years, prediction with data from only Olympic years between 1996 and 2021. Predictions of time under the current world record are reported in bold. M, male; and F, female.

When considering Olympic years, the 50, 100, 400, and 800 m front crawl swimming WRs are predicted to be broken for male (−0.55, −0.19, −0.03, and −0.50%, respectively; Table 3) but all WRs are predicted to hold for female (+2.16 ± 0.3%; Table 3).

## Discussion

The predictive model indicates a promising outlook for the 2024 swimming season. Expectations included notable advancements in female’s events, with potential WR breakthroughs in four out of six distances. For men, the focus shifted to middle distances, promising an exciting season of endurance and technical mastery.

The models showed remarkable precision in estimating front crawl swimming speeds from 1995 to 2023 for both male and female, with an average relative error of 0.56% ± 0.17%. This accuracy is below that found by Peronnet and Thibault (1989), which was 0.73% but calculated over only 1 year, and at the same level (0.57%) than the Bayesian time series regression recently employed by Wu et al. (2022).

When comparing predictive models for male, the Olympic model appeared more optimistic, projecting the breaking of four WRs (50, 100, 400, and 800 m), whereas all year’s model predicted to break two WRs (400 and 800 m). This discrepancy is not unexpected, given the inherent limitations of the Olympic model, which is based on only seven data points. Such a limited dataset risks a heightened influence from variations in individual data points. Furthermore, it is important to consider the broader context of athlete performance within a 4-year Olympic cycle. It has been observed that swimmer individual performance typically increases by 3%–4% during this period (Costa et al., 2011). This improvement can be attributed to various factors, including rigorous training regimens, strategic preparations leading up to the Olympic Games, and the intensity of competition during qualification events. Therefore, the observed discrepancies between the predictive models may also reflect the dynamic nature of athletic performance within the context of Olympic cycles and competitive events.

Regarding the male’s 400 m front crawl swimming WR set in 2009, this WR is at the time of writing still standing. The forecasted breach in 2024 is anticipated with optimism, given the emergent talents like Samuel Short and Ahmed Hafnaoui. Their personal bests, set in 2023, were remarkably close to the standing WR, with marginal differences of merely 0.61 and 0.63 s, respectively. This proximity underscores the potential for these young swimmers to surpass the current record. Foster et al. (2012) demonstrated that the introduction of full polyurethane swimsuits in 2009 significantly enhanced performance across distances ranging from 50 m to 1,500 m, with the greatest improvement observed in the 50 m freestyle. In longer swimming distances, the freestyle WR has remained unchanged since 2009, with no new records being set except for one instance in 2012 for the 1,500 m. This phenomenon has led to speculation that the stiffness of full-body polyurethane swimsuits may have adversely affected longer events. Additionally, the drag force experienced by swimmers, which is proportional to the square of the velocity, is reduced by these swimsuits. As a result, it is theorized that events with faster velocities, such as sprints, may benefit more from the use of such swimsuits. Additionally, research has shown that during the 400 m freestyle, aerobic metabolism contributes to 55% of energy output in the first 60 s, increasing to 95% between 60 and 190 s (Laffite et al., 2004). Consequently, one could hypothesize that distances under 200 m favor anaerobic capacity, often quantified in terms of peak power output (Lundsgaard et al., 2023), making strength and conditioning pivotal for shorter distances. The role of strength training in swimmers has been a subject of debate in the scientific community (Wirth et al., 2022). Therefore, it is conceivable that improvements in WRs for shorter distances since 2009 may be partly attributed, among other factors, to advancements in understanding and implementing strength and conditioning programs for swimmers.

Conversely, the scenario surrounding the 800 m front crawl swimming record appears more complex. The WR, also set in 2009—a time when full-body polyurethane swimsuits were permitted—poses a significant challenge. Although Short and Hafnaoui are identified as prodigies in swimming, their current times are still a few seconds shy of the WR. The notable gap in performance could partially be credited to the technological benefits offered by the full-body swimsuits of that period. Notably, in the same race, Tunisian swimmer Mellouli also surpassed the previous WR set by Grant Hackett in 2005. Presently, Hafnaoui’s time is 1.65 s faster than Hackett’s old record. Without the extraordinary feats achieved in 2009, this would currently stand as the WR.

In none of the models was the 1,500 m WR broken. This perhaps underlines a limitation of the present model, namely that it does not entirely consider technical developments, particularly those concerning turns. Indeed, turns averages 37% of the total race time (Morais et al., 2023) and it has been underlined that turns in 1,500 m freestyle race are essential for performance in elite swimmers (Polach et al., 2021).

For female, models have evolved in a different way, with the opposite trend to male. In fact, the all years model predicted WRs in the 50, 200, 400, and 800 m, while the Olympic model predicted no WR at all. This difference, which contradicted the previous reasoning for male, can be explained by the non-significant relationship of the two variables *K* and *n* as a function of time, resulting in using the mean value instead of extrapolation value in the Olympic model.

The comparison of the all year model between the two genders showed that female should perform more than male. Historically, female high performances observations lack almost 40 years of hindsight compared to male due to the later development of female’s sport. Consequently, the dynamic of improvement in female performance has been greater than male resulting in more ambitious prediction model for female (Busso and Thomas, 2006; Silva et al., 2007). The model seemed to be heading in that direction as MAP increased significantly for women over the years, while it remained unchanged for men, indicating ongoing enhancements in maximal oxygen consumption for women, in contrast to men. Moreover, unlike male, none of the female’s freestyle WR date back to 2008 or 2009. This suggests that the influence of body swimsuits does not have the same effect on female as it has on male. Issurin et al. (2014) examined the impact of high-tech swimsuits on 50-m performance. The authors observed a greater effect among male compared to female swimmers, particularly in freestyle and backstroke events.

In addition, averaging the years of each current record in female’s front crawls gave 2020 *versus* 2013 for male, underlining a more solid recent dynamic for female. Taken together, it could explain the relatively high number of predict WR of all year model for female.

The 200 and 400 m WRs established in 2023 are attributed to young female swimmers, potentially approaching their optimal performance years, generally identified between ages 21 to 26, with an anticipated peak performance duration of approximately 2.6 ± 1.5 years (Allen et al., 2014). The anticipated model forecasted slight enhancements in WRs for these disciplines, projecting an advancement of 0.19% for the 200 m and 0.029% for the 400 m. These projections were less ambitious than Seiler’s analysis (Hopkins, 2000), which estimated a decade progressivity rate of 1% for sprinting, 1.5% for distance running, and 5% for swimming.

In the case of the 50 and 800 m events, the situation is less clear-cut as the current WRs are held by athletes with extensive careers. However, in 2023, the iconic swimmer Sarah Sjöström managed to surpass the 50 m WR, showcasing her enduring capabilities despite her long-standing career. This achievement underscores her ability to continuously challenge boundaries. Conversely, the 800 m event presents a more intricate scenario. Since the establishment of the WR in 2016, the best performance to date was recorded in 2024 by Summer McIntosh, clocking in at 491.39 s. However, this time falls more than 6 s short of the current WR, highlighting the significant gap that remains to be bridged.

For both male and female swimmers, there remains ample scope for enhancement, particularly in the realms of resistance training and cross-training. The literature continues to grapple with delineating effective methods for transferring gains from resistance training to swimming performance, as well as determining optimal strategies for increasing training volume through cross-training modalities (Wirth et al., 2022). Additionally, the monitoring strategies for managing swimmer training loads continue to evolve, incorporating markers such as blood lactate levels and mood state profiling. These approaches aim to mitigate the risk of injury while optimizing performance (Feijen et al., 2020), potentially offering a competitive edge soon.

When assessing the model, it is crucial to recognize several limitations. First, the Olympic model lacks sufficient data, leading to the absence of linear relationship for the variables *K* and *n* with years for women. This observation aligns with previous research highlighting the challenge of modeling performance accurately with limited data. To address this, future studies could explore the incorporation of additional historical data or extend the analysis by including Olympic years before 1995. Seond, the model overlooks technical advancements in areas such as diving, turning, and starting, which can significantly influence performance outcomes (Figueiredo et al., 2020). Finally, the model was not individualized in terms of anthropometric characteristics, such as weight and height, of the record-setting athlete in any given year. Instead, the model represented two prototypical athletes (male and female) with standardized weight and body surface area. The age or biological age was also not considered in the present study, which might however be necessary for performance prediction in younger swimmers (Abbott et al., 2021). These limitations underscore the need for continued refinement and expansion of performance modeling approaches to better capture the complex dynamics of athletic performance.

To conclude, this investigation presents an innovative prognostic model for aquatic performance, integrating both historical outcomes and physiological metrics. This approach transcends conventional time-based forecasting by mitigating the impact of exogenous variables, such as the advent of full-body swimwear and disruptions caused by the COVID-19 pandemic, which have historically exerted a significant influence on the evolution of swimming performance. The inclusion of data derived from track and field athletics provides a robust foundation for this model, aiming to offer predictions less susceptible to these external perturbations. The results highlight a gender-specific divergence in performance trajectories, with forecasts showing a more optimistic outlook for female competitors, indicative of ongoing physiological progress in female’s swimming compared to a more stable trend observed in male. Moreover, the study suggests that relying solely on Olympic Games performances for predictions may be premature. With 15 new WRs set in 2023 all disciplines combined; this research underscores the prevailing competitive spirit within the swimming community. Looking ahead, this study anticipates that the trend of record-breaking performances will continue into 2024, underscoring the enduring dynamism and competitive intensity inherent in the sport.

## Data Availability

The datasets presented in this study can be found in online repositories. The names of the repository/repositories and accession number(s) can be found below: https://www.worldaquatics.com/swimming/records?recordCode&equals;WR&eventTypeId&equals;®ion&equals;&countryId&equals;&gender&equals;M&pool&equals;LCM.
